# Ventrolateral prefrontal cortex is required for performance of a strategy implementation task but not reinforcer devaluation effects in rhesus monkeys

**DOI:** 10.1111/j.1460-9568.2009.06740.x

**Published:** 2009-05

**Authors:** Mark G Baxter, David Gaffan, Diana A Kyriazis, Anna S Mitchell

**Affiliations:** Department of Experimental Psychology, Oxford UniversitySouth Parks Road, Oxford, OX1 3UD, UK

**Keywords:** macaque, monkey, prefrontal cortex, reinforcement, reward, rules

## Abstract

The ability to apply behavioral strategies to obtain rewards efficiently and make choices based on changes in the value of rewards is fundamental to the adaptive control of behavior. The extent to which different regions of the prefrontal cortex are required for specific kinds of decisions is not well understood. We tested rhesus monkeys with bilateral ablations of the ventrolateral prefrontal cortex on tasks that required the use of behavioral strategies to optimize the rate with which rewards were accumulated, or to modify choice behavior in response to changes in the value of particular rewards. Monkeys with ventrolateral prefrontal lesions were impaired in performing the strategy-based task, but not on value-based decision-making. In contrast, orbital prefrontal ablations produced the opposite impairments in the same tasks. These findings support the conclusion that independent neural systems within the prefrontal cortex are necessary for control of choice behavior based on strategies or on stimulus value.

## Introduction

Adaptive behavior requires capacities beyond simple stimulus–response learning. The ability to make decisions based on complex behavioral strategies and rules provides for a much richer behavioral repertoire in response to changing environmental demands, as well as more efficient accumulation of rewards. The prefrontal cortex is heavily implicated in strategic and decision-making behavior. It contains a number of subregions that can be differentiated based on cytoarchitectonics, chemoarchitectonics and anatomical connections (Mrzljak & Goldman-Rakic, 1992; Carmichael & Price, 1994, 1995a,b; Petrides & Pandya, 1999, 2002). There is general agreement about the importance of the prefrontal cortex for strategic behavior. However, little is known about the extent to which different subregions of the prefrontal cortex are required for specific aspects of decision-making, based on impairments of particular aspects of behavior by focal damage to subregions of the prefrontal cortex. Such data would facilitate the discovery of the neural mechanisms by which information about stimulus identity and value is integrated with biological drives in order to adaptively control behavior.

The present study was designed to elucidate the involvement of the ventrolateral prefrontal cortex (VLPFC), composed of areas 47/12 and 45A (Petrides & Pandya, 2002), in decision-making functions attributed to the prefrontal cortex. VLPFC is densely connected with inferior temporal cortical areas involved in processing of information about visual objects (Webster *et al.*, 1994; Petrides & Pandya, 2002), and is associated both with the representation of rule information associated with visual cues (Bunge *et al.*, 2003) and with the storage of sequences of information in working memory (Owen *et al.*, 1999). We hypothesized that the VLPFC specifically may be required for efficient performance of a strategy implementation task in which different categories of objects are associated with different strategies for obtaining reward (Gaffan *et al.*, 2002), which requires representation of behavioral rules associated with different categories of stimuli as well as appropriate sequencing of choice behavior in response to changing demands of the task. Performance of this task requires intrahemispheric interaction between frontal and inferotemporal cortex (Gaffan *et al.*, 2002), but does not require an intact orbital or dorsolateral prefrontal cortex (Baxter *et al.*, 2007, 2008b).

The VLPFC is also implicated in goal-directed behavior (Sakagami & Pan, 2007). The strategy implementation task requires the use of rules in order to guide choice behavior, but does not require the representation of specific goals because all rewards earned are the same regardless of the rules employed to obtain them. Thus, we also examined the involvement of the VLPFC in value-based decision-making using a reinforcer devaluation procedure. Normal monkeys make appropriate adjustments to their choice behavior in this task in response to a change in the value of a particular reinforcer, but monkeys with lesions of the amygdala or orbital prefrontal cortex do not (Málková*et al.*, 1997; Baxter *et al.*, 2000; Izquierdo *et al.*, 2004; Machado & Bachevalier, 2007). This is in spite of their ability to make appropriate choices when confronted with the reinforcers directly (Izquierdo *et al.*, 2004; cf. Machado & Bachevalier, 2007). The interconnectivity of the VLPFC with the orbital prefrontal cortex (Carmichael & Price, 1996; Petrides & Pandya, 2002) would support the involvement of the VLPFC in value-based decision-making as well.

## Materials and methods

### Subjects

Eight rhesus monkeys (*Macaca mulatta*), six male (CON1/VL3, CON2, CON4, VL1, VL2, VL4) and two female (CON3, CON5), 3.33–7.44 kg (22–51 months old) at the beginning of behavioral training, participated in this study. The monkeys were housed socially in troops, separated by sex, in indoor enclosures attached to standard caging. Water was always available *ad libitum* in the home enclosure; each monkey’s daily food ration was delivered in the test box and was supplemented with fruit and forage mix in the home enclosure. All procedures were conducted under the authority of UK Home Office personal and project licences held by the authors.

All monkeys were preoperatively trained on a scene-learning task (Gaffan, 1994; Baxter *et al.*, 2007), the results of which are described elsewhere (Baxter *et al.*, 2007, 2008a), and on the strategy implementation task. They then received a preoperative performance test in which sessions of both tasks were interleaved with one another. Three monkeys (VL1, VL2, VL4) then received bilateral VLPFC lesions, and four (CON1, CON2, CON3, CON4) were retained as unoperated controls. The eighth (CON5) received bilateral injections of sterile saline into the VLPFC bilaterally, as a control case for a separate experiment on neurochemical lesions of the VLPFC. They then received a postoperative performance test of scene learning and strategy implementation, followed by training on object–reward association learning and concurrent and serial reversal learning (the results of which are not discussed here), and finally reinforcer devaluation. At this point one monkey (CON1) received a further performance test of scene learning and strategy implementation (given in the same way as the other performance tests) and received a VLPFC lesion, then had a postoperative performance test in scene learning and strategy implementation, and a retest in reinforcer devaluation (based on a new set of object discrimination problems). Data from his performance in this second phase of testing are designated as coming from case VL3, although this is the same monkey as case CON1.

With regard to the sequence of preoperative training, four of the monkeys (VL1, CON2, VL2, CON1/VL3) underwent pretraining and then learned several two-choice visual discrimination problems in a touchscreen apparatus (Baxter & Gaffan, 2007) before beginning training on the strategy implementation task; they then acquired the scene memory task. The other four monkeys underwent pretraining, then learned the scene memory task followed by the strategy implementation task. Critically, the preoperative and postoperative performance tests were identical for all eight monkeys.

A fifth monkey with intended bilateral damage to the VLPFC was tested as part of this experiment, but she had unintended damage to the lateral orbital prefrontal cortex bilaterally (case VL5; Baxter *et al.*, 2008a). She was impaired on both of the tasks discussed in this paper (strategy implementation and reinforcer devaluation), so her performance is consistent with the conclusions about the functions of these cortical areas; but because her data do not elucidate the dissociation of function between VLPFC and orbital prefrontal cortex they are not reported here.

We contrasted the results from these monkeys with results from a group of monkeys with orbital prefrontal ablations (cases ORB1–ORB3), tested in precisely the same way as the present experiment. These ablations were without effect on strategy implementation (Baxter *et al.*, 2007). We expected that the orbital ablation would produce a severe impairment in reinforcer devaluation, in view of previous evidence for the involvement of the orbital prefrontal cortex in this task (Izquierdo *et al.*, 2005; Machado & Bachevalier, 2007). Effects of VLPFC lesions on performance of this task have not been assessed before. These monkeys were tested in precisely the same fashion as the monkeys with VLPFC lesions.

### Apparatus

Behavioral testing took place in an automated apparatus. Each monkey was taken from the home enclosure into the test cubicle in a wheeled transport cage, which was fixed in front of a video-display unit with a touch-sensitive screen (380 × 280 mm, 800 × 600 pixel resolution). The monkey could reach through horizontally oriented bars (approximately 45 mm apart) at the front of the cage to reach the screen and the rewards. Stimulus presentation, recording of touches to the screen and reward delivery were all under computer control. A pellet dispenser delivered 190-mg banana-flavored or sugar pellets (P. J. Noyes, Lancaster, NH, USA) into a food cup located below the touchscreen. Pellet delivery produced a click from the pellet dispenser as well as a 500-ms tone from the computer. A metal ‘lunchbox’ (approximately 200 × 100 × 100 mm) was located to the left of the food cup and was filled with a mixture of wet monkey chow, seeds, apple, banana, orange, nuts and dates. This lunchbox contained the ‘large food reward’ that was delivered at the completion of testing. Infrared cameras positioned at different locations within the test cubicle permitted observation of the monkey while it was performing the task. The entire apparatus was located in an experimental cubicle that was dark except for the illumination of the video screen.

### Behavioral testing: pretraining

The monkeys that had experience with a discrimination learning task in the touchscreen had no further pretraining before beginning training on the strategy implementation task (described in the next section). The remaining monkeys were shaped to enter the transport cage from their home enclosure, and once they were reliably taking food in the test cubicle, pretraining began. First, reward pellets were delivered on a variable-interval (2-min) schedule to accustom them to take pellets in the test box. After several days of pellet training, the touchscreen was activated and the screen was filled with an array of different-colored alphanumeric characters on a black background (in a different size and typeface than those used in the main task). Touches to any location on the screen resulted in pellet delivery. In the third stage, single alphanumeric characters were presented in random locations on the screen, and remained until touched; a touch caused the character to disappear and a reward pellet to be delivered. Gradually, the complexity of the display was increased by introducing additional visual elements (a colored background, colored ellipse segments and a single large alphanumeric character). When monkeys were reliably completing 50 trials in a single test session with minimal accuracy errors (i.e. touching any location on the screen other than the small alphanumeric character) they began training on the scene memory task. The monkeys with discrimination learning experience underwent this third stage of pretraining between acquisition of the strategy task and the scene task.

### Strategy implementation task

This task is identical to that described by Gaffan *et al.* (2002), except that clip art stimuli were used instead of compound alphanumeric characters. In this task, monkeys learned about four pairs of clip art stimuli, which were used in all pre- and postoperative testing on this task. The task is a form of conditional discrimination learning that requires monkeys to learn to execute sequences of choices from two arbitrarily defined categories of objects, each associated with a different behavioral strategy. One stimulus in each pair was associated with a ‘persistent’ strategy (P), the other was with a ‘sporadic’ strategy (S). These ‘strategies’ refer to the patterns of choice behavior in the task that could lead to reward. Four consecutive persistent choices resulted in a reward after the fourth choice; any time after that a sporadic choice was rewarded immediately, but sporadic choices were not rewarded again until another persistent reward had been earned. Thus, optimal performance in this task was achieved by alternating categories of object choices upon receiving reward: make four consecutive P choices, obtaining a reward on the fourth choice, then choose S once, obtaining a reward immediately, then return to choosing P until a reward is earned again, etc. Each pair of stimuli was learned individually, then they were presented randomly intermixed within the test session so that performance had to be guided by the strategy associated with each object rather than a specific sequence of choices of individual objects. Thus, in the final version of the task, there was no single discrete conditional cue that signaled the appropriate action; good performance depended on linking objects to strategies and organizing a sequence of choices (Gaffan *et al.*, 2002).

A pair of objects appeared on the touchscreen on each trial, containing one object from each category, and the monkey was allowed to choose one of the two objects. The left–right position of the objects on the screen was randomized across trials. After one of the two objects was touched, the screen blanked for a 5-s intertrial interval before the next trial was presented. Monkeys could earn rewards in one of two ways. First, four consecutive choices of the ‘persistent’ object within each pair resulted in delivery of a 190-mg pellet upon the fourth persistent choice. Second, any time after receiving a reward for choosing four persistent objects in a row, a single choice of an object from the second category (‘sporadic’) resulted in banana pellet delivery, but another sporadic reward was not given until another persistent reward had been earned. The dependent measure was the trials/reward ratio. The choice sequence that would optimize the rate of reward delivery was for the monkey to choose the persistent object in the pair on four consecutive trials, then the sporadic object on the following trial, and then to repeat this sequence of choices, resulting in two rewards for every five trials (a trials/reward ratio of 2.5). Failing to choose the sporadic object immediately after receiving a reward for choosing four persistent objects in a row, interrupting chains of persistent responses with choices of sporadic objects, or continuing to choose the sporadic object before another reward had been earned for choosing persistent objects all contributed to less-than-optimal performance and an elevation of the trials/reward ratio. In each test session, monkeys chose objects across trials until they had earned 50 rewards. (Postoperatively, VL1 and VL4 were given sessions in which they only had to earn 30 rewards, to prevent frustration in completing the test sessions; similar to Gaffan *et al.*, 2002.) The final rewarded trial of each session resulted in delivery of the large food reward in the ‘lunchbox’ as well as the final reward pellet.

Training procedures were identical to Gaffan *et al.* (2002) and proceeded in five phases. Briefly, monkeys were trained on this task by presenting one pair of objects at a time (containing one persistent object and one sporadic) until the trials/reward ratio was 2.94 or lower in each of two consecutive sessions in which 50 total rewards were earned, or until a total of 6000 (first problem) or 4000 (all other phases) rewards had been earned. Once this criterion was achieved with each pair individually, in the fifth and final phase (the final version of the task) the four pairs of objects were presented randomly intermixed across trials so that choice behavior had to be guided by the category membership of each object rather than a sequence of specific object choices. Training in this phase continued to the same criterion (two consecutive sessions with a ratio of 2.94 or better or 4000 rewards earned, about 80 sessions of training). Choice behavior was above chance in the first session with intermixed problems, mean trials/reward ratio = 4.15; chance performance would be 16.3 (Gaffan *et al.*, 2002). Monkeys that did not reach the 2.94 trials/reward criterion and advanced based on the cumulative number of rewards earned within a phase (VL2, second problem, CON2, third problem and final phase, CON4 and CON5, final phase) performed comparably in their preoperative performance test to other monkeys that had achieved the criterion during training. For all eight monkeys, the mean number of sessions required to complete all five phases of training was 186.9 (range 80–414); to complete the final phase of training it was 51.5 (range 7–149).

### Performance tests

After completion of training on the scene-learning and strategy tasks, all monkeys were given a preoperative performance test consisting of 24 sessions. Testing of performance in the strategy implementation task was interleaved with sessions in which new object-in-place scene problems were learned (Baxter *et al.*, 2007). The first session was scene learning, followed by five cycles of two sessions of strategy performance followed by two sessions of scene learning, then two sessions of strategy performance, then a final session of scene learning. The sequence of sessions was thus STTSSTTSSTTSSTTSSTTSSTTS, where ‘S’ represents a session of scene learning and ‘T’ represents a session of strategy implementation testing. Data from the first four sessions were not considered (to allow monkeys to become accustomed to the alternating tasks), leaving 20 sessions of performance data (10 of scene learning, 10 of strategy implementation). In this design we could compare performance on each task when it was preceded by performance on the same or a different task, although we did not observe any systematic variation in performance related to this variable either before or after surgery. This test was repeated in the same way beginning at least 2 weeks after surgery (for monkeys in the VLPFC group) or an equivalent period of rest for control monkeys.

### Surgery

Neurosurgical procedures were performed in a dedicated operating theater under aseptic conditions. Cases VL1–VL4 received a bilateral ablation of the VLPFC. In cases VL2–VL4, steroids (methylprednisolone, 20 mg/kg) were given i.m. the night before surgery, and three doses were given 4–6 h apart (i.v. or i.m.) on the day of surgery, to protect against intraoperative edema and postoperative inflammation. Case VL1 received dexamethasone (2 mg/kg) i.v. once during the surgery only. Each monkey was sedated on the morning of surgery with both ketamine (10 mg/kg) and xylazine (0.5 mg/kg), i.m. Once sedated, the monkey was given atropine (0.05 mg/kg) to reduce secretions, antibiotic (amoxicillin, 8.75 mg/kg) for prophylaxis of infection, opioid (buprenorphine 0.01 mg/kg i.v., repeated twice at 4–6-h intervals on the day of surgery, i.v. or i.m.) and non-steroidal anti-inflammatory (either meloxicam, 0.2 mg/kg, i.v. or carprofen, 4 mg/kg, i.m.) agents for analgesia, and an H2 receptor antagonist (ranitidine, 1 mg/kg, i.v.) to protect against gastric ulceration as a side-effect of the combination of steroid and non-steroidal anti-inflammatory treatment. The head was shaved and an intravenous cannula put in place for intraoperative delivery of fluids (warmed sterile saline drip, 5 mL/h/kg). The monkey was moved into the operating theater, intubated, placed on isoflurane (VL1, VL3, 1–2.5%, to effect, in 100% oxygen) or sevoflurane (VL2 and VL4, 2.25–4.5%, to effect, in 100% oxygen) anesthesia, and then mechanically ventilated. Adjustable heating blankets allowed maintenance of normal body temperature during surgery. Heart rate, oxygen saturation of hemoglobin, mean arterial blood pressure, end tidal CO_2_, body temperature and respiration rate were monitored continuously throughout surgery.

The monkey was placed in a head-holder and the head cleaned with alternating antimicrobial scrub and alcohol and draped to allow a midline incision. The skin and underlying galea were opened in layers. The temporal muscles were retracted as necessary to expose the skull surface over the intended lesion site. A bone flap was turned over the frontal lobes and the craniotomy was extended with rongeurs as necessary. The dura was cut and reflected over the frontal lobes. The VLPFC was removed bilaterally extending from the ventral lip of the principal sulcus to the fundus of the lateral orbital sulcus. The anterior limit was a line joining the anterior tips of the principal and lateral orbital sulci. The posterior limit was a line joining the posterior tip of the principal sulcus and the anterior tip of the inferior limb of the arcuate sulcus, then extending from the tip of the arcuate sulcus to the posterior tip of the lateral orbital sulcus. All of the cortex was removed within these limits. Cortical tissue was removed by subpial aspiration using a small-gage sucker insulated everywhere except at the tip; electrocautery was applied to remove the pia mater and control bleeding encountered during the ablation.

When the lesion was complete, the dura was sewn over the lesion site, the bone flap replaced and held with loose sutures, and the skin and galea were closed in layers. The monkey was removed from the head-holder and anesthesia discontinued. The monkey was extubated when a swallowing reflex was observed, returned to the home cage, and monitored continuously until normal posture was regained (usually within 10 min). Non-steroidal anti-inflammatory analgesic (meloxicam, 0.2 mg/kg, oral) and antibiotic (amoxicillin, 8.75 mg/kg, oral) treatment continued following surgery in consultation with veterinary staff for 4–5 days. Operated monkeys rejoined their social groups as soon as practicable after surgery, usually within 3 days of the operation.

Surgical procedures for cases ORB1–ORB3 are described in a previous publication (Baxter *et al.*, 2007) but are similar to those for cases VL1–VL4, except for the extent of the intended lesion. Case CON5 underwent similar surgical procedures to cases VL1–VL4, but received a total of 34 (17 per hemisphere) handheld 1-μL sterile phosphate-buffered saline injections into The VLPFC, placed approximately 3 mm apart. The boundaries for these injections were the same as those for the VLPFC ablation: they extended from the ventral lip of the principal sulcus to the fundus of the lateral orbital sulcus. The anterior and posterior limits were lines joining the tips of the principal and lateral orbital sulci.

### Reinforcer devaluation testing

This task followed procedures described by Málková*et al.* (1997) and Baxter *et al.* (2000), except that it took place in an automated apparatus instead of a manual one. Sixty pairs of clip-art objects were used, each pair constituting a problem. One of the two clip-art objects was arbitrarily designated correct in each pair. The objects were presented against a gray background, one on the left side of the screen and one on the right, which was randomized across trials. Touching the correct object resulted in the incorrect object disappearing, delivery of a reward, then the correct object disappearing after 1 s. Touching the incorrect object caused both objects to disappear immediately and no reward was delivered. All 60 problems were given in a single block in each session, in the same order for each session. The intertrial interval was 30 s regardless of whether the choice was correct or incorrect. Half of the rewarded objects resulted in the delivery of a half-peanut, and the other rewarded objects produced an M&M chocolate candy. As in the strategy implementation task, monkeys received a large food reward at the end of each test session, but it was handed to the monkey at the end of the session, rather than being given from the metal lunchbox.

Training continued with daily sessions of the 60 problems until a criterion of 270 or more correct responses over five consecutive sessions (90% or greater correct) was reached. At this point a series of sessions of critical trials was presented in which the 60 rewarded objects were randomly reassigned to pairs to create 30 critical trials, each offering a choice between a peanut-rewarded object and an M&M-rewarded object. Thus, a critical trial session comprised 30 trials, with each rewarded object appearing only once in each session, but was otherwise identical to the standard training sessions: rewards were delivered after object choices (and because each trial was composed of a choice between two rewarded objects, a reward was earned on each trial). Different random pairings of objects were used for each critical trial session.

Reinforcer devaluation effects were tested in a series of critical trial sessions. In each series, two of these sessions served as baseline sessions and were not preceded by any special treatment. Two further sessions were conducted preceded by selective satiation of one of the two foods. For the devaluation, the monkey was moved into the transport cage and remained in the housing room. A plastic box was affixed to the front of the cage containing a known amount of food reinforcer (either M&Ms or peanuts). The monkey was left undisturbed for 15 min to consume the food. If the food was completely eaten the box was refilled. The monkey was then observed closely, and once it had not taken any food for 5 min the box was removed from the cage. Once the monkey’s cheek pouches were not visibly full of food, it was moved to the testing cubicle and the critical trial session begun. The sequence of critical trial sessions was: baseline, peanut devaluation, baseline, M&M devaluation. This series was repeated once. Each critical trial session was separated by at least one standard training session, and monkeys had at least two days of rest following a critical trial session in which devaluation occurred.

The critical measure was a score composed of the difference in number of choices of objects paired with a particular food on baseline sessions and in sessions when that food was devalued. These scores were added together for each devalued food. This was calculated separately for each sequence of critical trial sessions and the mean taken as the overall score. For example, a monkey that chose 12 M&M objects and 18 peanut objects in the baseline sessions (mean of the two baseline sessions), then chose five peanut objects when peanuts were devalued and seven M&M objects when M&Ms were devalued would have a difference score of (18−5) + (12−7) = 18. If he chose 14 M&M objects and 16 peanut objects in baseline sessions of the second set of critical trial sessions, then three peanut objects and seven M&M objects when each was devalued, this would give a score of 20 for the second set of critical sessions and a difference score of 19 overall.

CON3 would not take the half-peanut rewards in the test apparatus, so was therefore not tested in reinforcer devaluation. Case VL2 showed an extreme preference for M&Ms and would not select peanut objects in any of the first four critical trial sessions, so his data were discarded. Note that case CON1/VL3 was tested in devaluation twice; after his VLPFC lesion, a new set of 60 object discrimination problems was learnt for assessment of the effect of his lesion on devaluation.

### Histology

**Fig. 1:**
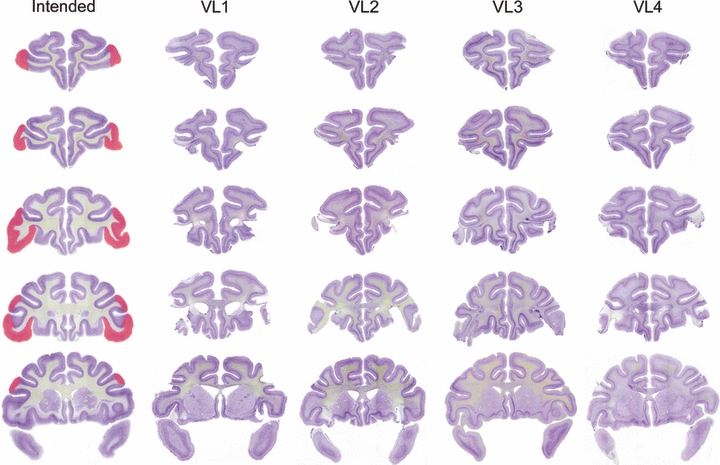
Lesions of VLPFC in cases VL1–VL4. The intended lesion is shown in red on coronal sections of a normal rhesus monkey in the leftmost column, at five stereotaxic levels through the prefrontal cortex, moving from rostral to caudal from the top to the bottom of the figure. Photomicrographs of brain sections from cases VL1–VL4 at corresponding stereotaxic levels are shown in the other columns.

After completion of behavioral training each operated monkey was sedated with ketamine (10 mg/kg), deeply anesthetized with i.v. barbiturate and transcardially perfused with 0.9% saline followed by 10% formalin. The brain was cryoprotected in formalin-sucrose and then sectioned coronally on a freezing microtome at 50 μm thickness. A 1-in-10 series of sections through the area of the lesion was mounted on gelatin-coated glass microscope slides and stained with Cresyl violet. Lesions are illustrated in Fig. 1.

### Data analysis

Non-parametric statistics (Kruskal–Wallis *H*-tests or Mann–Whitney *U*-tests for between-groups comparisons and Wilcoxon signed-ranks tests for within-group comparisons) were used for all analyses, because of variability within the VLPFC lesion group. The exception to this was the analysis of strategy behavior (probability of sporadic choices after different numbers of persistent choices), which required parametric anova for evaluation of higher-order interaction effects that cannot be examined with non-parametric tests. A focused contrast in the overall anova evaluated the three-way interaction of number of persistent choices, pre/post, and lesion group, based on the hypothesis that strategy implementation deteriorates postoperatively because of an increase in inappropriate sporadic choices (after one, two or three persistent choices) and a decrease in appropriate sporadic choices (after four persistent choices).

## Results

### Extent of VLPFC lesions

**Fig. 2:**
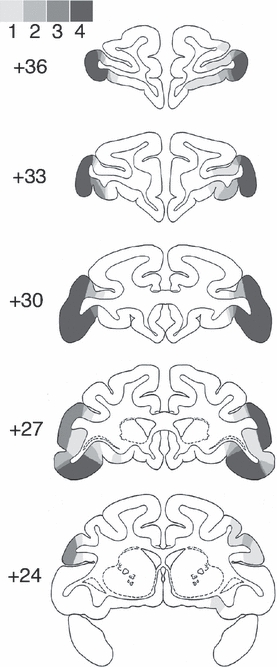
The extent of VLPFC damage is shown on coronal sections from a standard rhesus monkey brain atlas (Szwarcbart, 2005) at five stereotaxic levels through the frontal lobes (numerals represent mm anterior to the interaural plane) for cases VL1–VL4. Areas of lesion overlap are illustrated in shades of gray, the darkest indicating damage present in all four cases, the lightest indicating damage present in only one of the four cases.

**Fig. 3:**
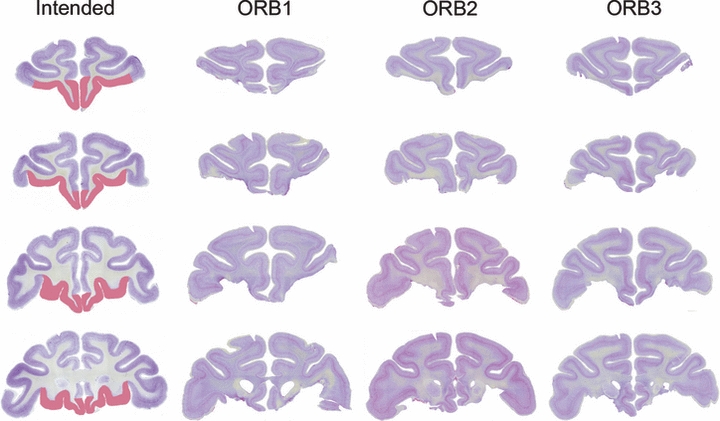
Lesions of orbital prefrontal cortex in cases ORB1–ORB3. The intended lesion is shown in red on coronal sections of a normal rhesus monkey in the leftmost column, at four stereotaxic levels through the prefrontal cortex, moving from rostral to caudal from the top to the bottom of the figure. These correspond to the approximate stereotaxic levels illustrated in Fig. 1 for the cases with VLPFC lesions. Photomicrographs of brain sections from cases ORB1–ORB3 at corresponding stereotaxic levels are shown in the other columns.

**Fig. 4:**
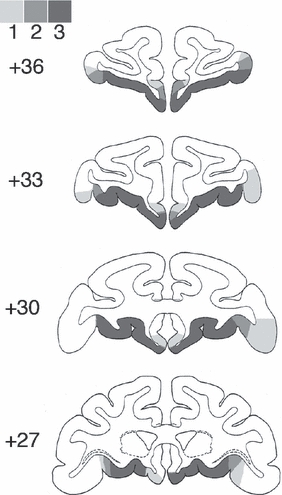
The extent of orbital prefrontal cortex damage is shown on coronal sections from a standard rhesus monkey brain atlas (Szwarcbart, 2005) at four stereotaxic levels through the frontal lobes (numerals represent mm anterior to the interaural plane). Areas of lesion overlap are illustrated in shades of gray, the darkest indicating damage present in all three cases, the lightest indicating damage present in only one of the three cases.

The VLPFC lesions were intended to damage areas 47/12 and 45A of Petrides & Pandya (2002), as well as the ventral portion of areas 9 and 46 ventral to the principal sulcus. They were intended to spare the dorsolateral prefrontal cortex (areas 9 and 46, in the banks of the principal sulcus and dorsal to it) as well as the cortex on the orbital surface (areas 11, 13 and 14). The lesions were similar in all four cases and relatively complete. Figure 1 shows photomicrographs from an unlesioned rhesus monkey brain, on which the intended lesion extent is indicated by red coloring, and sections from each lesion case VL1–VL4 at corresponding stereotaxic levels. In all four cases the lesions removed the VLPFC bilaterally. The lesion was slightly smaller in the anterior–posterior extent in case VL3 relative to the other three cases. The extent of damage outside this region was limited, although some damage to the anterior lateral orbital cortex was noted bilaterally in cases VL1 and VL2. Plots of VLPFC lesion overlap on drawings of a standard rhesus monkey brain (Szwarcbart, 2005) are illustrated in Fig. 2. Corresponding figures illustrating the orbital prefrontal lesions (cases ORB1–ORB3, which have been published previously; Baxter *et al.*, 2007) are shown in Figs 3 and 4. These lesions were intended to ablate the cortex on the orbital surface lateral to the fundus of the lateral orbital sulcus (areas 11, 13 and 14; Petrides & Pandya, 2002). Slight damage to the rostral VLPFC is present in these three monkeys, but again the lesions are similar in all three cases and complete.

### Strategy implementation

**Fig. 5:**
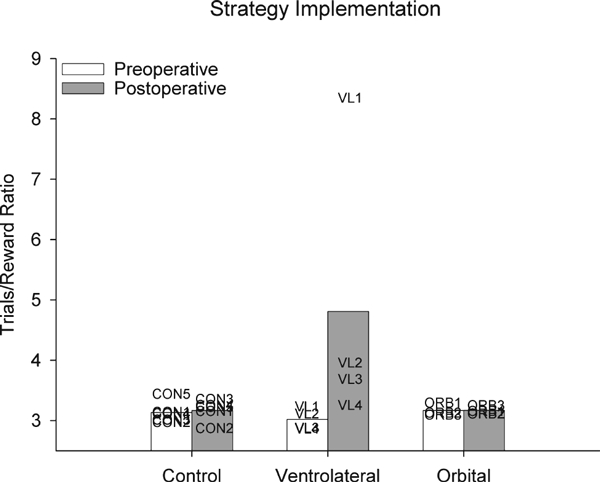
VLPFC lesions impair strategy implementation performance. The critical measure of strategy implementation performance (trials/reward ratio) is illustrated; a ratio of 2.5 represents perfect performance, and higher ratios reflect progressively worse performance. Open bars represent mean preoperative performance, gray bars represent mean postoperative performance, and the labels CON1–CON5, ORB1–ORB3 and VL1–VL4 represent scores of individual monkeys.

VLPFC lesions impaired performance of the preoperatively learned strategy implementation task, indicating an impairment in making decisions (choosing one of two objects on each trial) based on behavioral strategies. To compare the effects of lesions, the difference in performance (trials/reward ratio) between preoperative and postoperative tests was calculated for each monkey. A trials/reward ratio of 2.5 indicates perfect performance of the strategy implementation task (earning two rewards for every five trials worked); higher scores indicate deviations from optimal behavior. The decrement in postoperative performance in the VLPFC group was significantly larger than that of the control group, Mann–Whitney *U*=0, *P*=0.014. We also compared their performance to monkeys with bilateral ablations of orbital prefrontal cortex, which do not impair this task (Baxter *et al.*, 2007). The postoperative impairment in the strategy implementation task is significantly greater in the VLPFC group relative to monkeys with ablation of the orbital prefrontal cortex (who are unimpaired), Mann–Whitney *U*=0, *P*=0.034. These data are illustrated in Fig. 5, which displays the pre- and postoperative performance scores for each monkey.

**Fig. 6:**
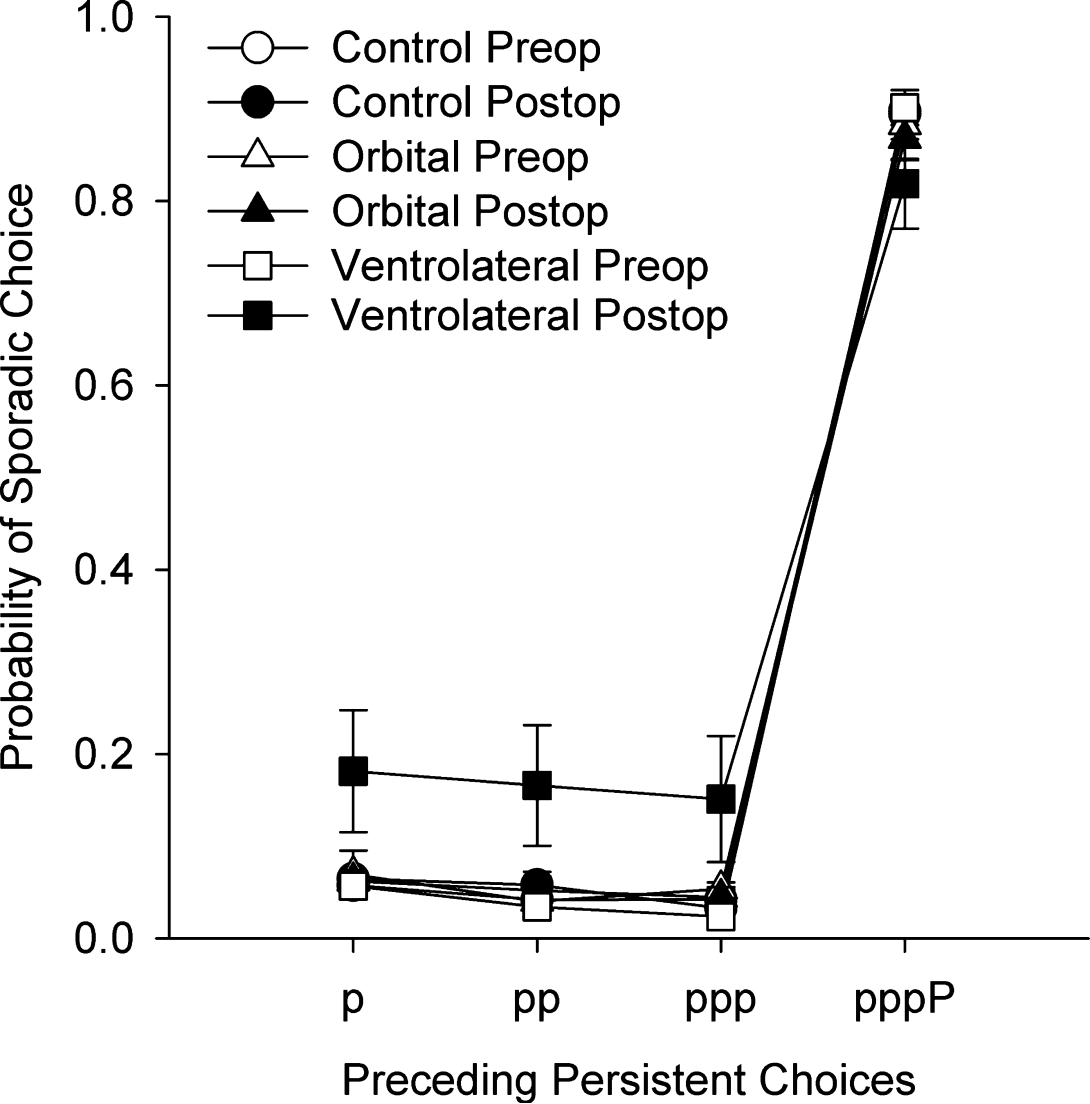
Probability of a sporadic choice after one, two, three or four persistent choices in control, VLPFC and orbital groups. Preoperatively in all groups, and postoperatively in control monkeys and monkeys with orbital prefrontal lesions, the probability of a sporadic choice after one, two or three persistent choices is extremely low, and it is very high after four consecutive persistent choices. This is consistent with correct and efficient application of the behavioral strategies associated with the different categories of objects. Postoperatively in the VLPFC group, the probability of making an inappropriate sporadic response after fewer than four persistent responses is increased, but the probability of making an appropriate sporadic response after four persistent responses is reduced. Thus, they are impaired in the overall implementation of behavioral strategies, and are not simply more likely to commit an impulsive sporadic response overall. Error bars indicate mean ± SEM.

We considered the nature of impairment in strategy implementation caused by the VLPFC lesion. Monkeys could perform poorly in this task for two reasons: they could make inappropriate sporadic choices that interrupt sequences of persistent choices that are necessary to gain rewards (and to enable a sporadic choice to give reward); and they could fail to make sporadic choices when they are appropriate (immediately after a rewarded persistent response). Frontal-inferotemporal disconnection causes both of these kinds of behavioral disruption (Gaffan *et al.*, 2002). We analysed the probability of a sporadic response after one, two, three or four consecutive persistent responses in each group of monkeys. For this measure, the probability of making a sporadic response after one, two, three or four persistent choices was determined for each test session for each monkey, and a mean value calculated for each monkey for its pre- and postoperative performance tests. Monkeys with VLPFC lesions performed more poorly than control monkeys in both aspects of strategy implementation, making more sporadic responses than controls after one, two or three consecutive persistent responses, and fewer sporadic responses than controls after four consecutive persistent responses (Fig. 6). Parametric anova revealed a critical three-way interaction of test phase (pre/post), lesion group and preceding number of persistent responses, *F*_6, 27_ = 3.13, *P*=0.018. This is consistent with less strategic and more random choice behavior postoperatively, rather than a shift in strategy (e.g. always selecting the persistent stimulus on every trial) that would also increase the ratio measure, but would suggest a different underlying behavioral impairment. A focused contrast decomposed this interaction, based on the increase in inappropriate strategic behavior postoperatively (an increase in inappropriate sporadic responses and a decrease in appropriate sporadic responses). This was calculated as a difference score between the increase in inappropriate sporadic responses (mean pre–post difference in probability of sporadic choice after one, two or three persistent responses) and decrease in appropriate sporadic responses (pre–post difference in probability of sporadic choice after four persistent responses). This comparison, using a pooled error term, revealed a significant difference between control and VLPFC monkeys, *t*_7_ = −2.35, *P*=0.025 (one-tailed), and between monkeys with VLPFC and orbital lesions, *t*_6_ = −2.00, *P*=0.043 (one-tailed).

### Reinforcer devaluation

**Fig. 7:**
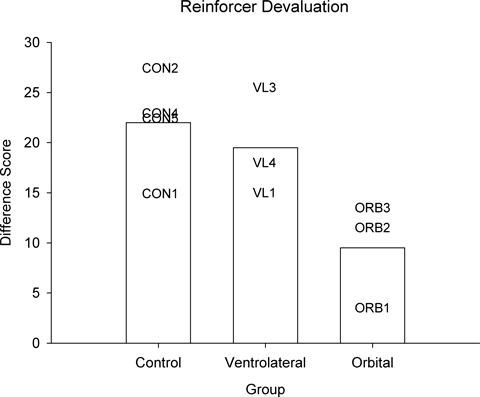
Performance in the reinforcer devaluation task is unimpaired in monkeys with ventrolateral prefrontal lesions, but it is impaired in monkeys with bilateral ablation of the orbital prefrontal cortex with identical testing histories (Baxter *et al.*, 2007) and tested in precisely the same procedures.

Monkeys with VLPFC lesions, in contrast to impaired performance in the strategy implementation task, showed intact reinforcer devaluation effects. There was no difference in the number of sessions to criterion for learning the discrimination problems (control mean 19.25, range 12–27; VLPFC mean 14.0, range 11–16; orbital mean 11.33, range 9–14), Kruskal–Wallis *H* = 3.27, *P*=0.19. The three groups of monkeys spent comparable amounts of time in the devaluation procedure (control, 27.8 min; VLPFC, 34.8 min; orbital, 30.1 min) and ate on average the same amount of food during the procedure (control, 122.2 g; VLPFC, 114.1 g; orbital, 93.6 g); Kruskal–Wallis tests confirmed the absence of significant differences between the groups on these measures (*H*s < 2.46, *P*>0.29). The VLPFC and control groups did not differ in reinforcer devaluation performance measured by the mean difference score across the two sets of devaluation tests, Mann–Whitney *U*=7.5, *P*=0.59 (Fig. 7). Monkeys with orbital prefrontal lesions were impaired relative to controls in devaluation performance in the critical trial sessions, Mann–Whitney *U*=12, *P*=0.034. There was also a significant difference between the VLPFC and orbital prefrontal lesion groups, *U*=0, *P*=0.05.

We analysed performance during the devaluation sessions and found no tendency of any group to change its responding over the course of the session; that is, monkeys were as likely to choose objects associated with the devalued food at the end of each session as at the beginning (data not shown). Thus, monkeys do not learn to avoid objects associated with the devalued reinforcer over the course of a critical trial session, so an impairment in devaluation in monkeys with orbital prefrontal lesions cannot be ascribed to a failure to exhibit this behavior.

## Discussion

Bilateral ablations of VLPFC impaired the performance of a preoperatively learned strategy implementation task. These ablations had no effect, however, on reinforcer devaluation effects. The opposite pattern of impairments was seen after orbital ablations: these had no effect on strategy implementation (Baxter *et al.*, 2007) but impaired reinforcer devaluation. These data demonstrate a double dissociation of function between VLPFC and orbital prefrontal cortex. Monkeys with VLPFC ablation are significantly impaired in strategy implementation relative to both unoperated controls and monkeys with orbital prefrontal ablation (who are not impaired relative to controls). In contrast, monkeys with orbital prefrontal ablation are impaired in reinforcer devaluation relative to both unoperated controls and monkeys with VLPFC ablation (who are not impaired relative to controls). The present data indicate that different cortical fields within the prefrontal cortex are necessary for different cognitive functions that rely on an intact prefrontal cortex. Previous evidence to this effect is sparse (Gaffan, 2002).

The current results show that VLPFC lesions impair performance of a preoperatively learned strategy implementation task that requires monkeys to alternate choices between two categories of visual stimuli in order to earn rewards at an optimal rate. It is notable that performance of this task is unaffected by lesions of either orbital (Baxter *et al.*, 2007) or dorsolateral prefrontal cortex (Baxter *et al.*, 2008b). This is consistent with a role for the VLPFC in representing sequences of behaviorally relevant stimuli or actions (Owen *et al.*, 1999; Shima *et al.*, 2007), in switching between cognitive sets (Nakahara *et al.*, 2002), as well as in representing semantic or categorical information associated with these stimuli (Bunge *et al.*, 2003; Gifford *et al.*, 2005; Cohen *et al.*, 2007), all capacities that may be engaged by the strategy implementation task. It is important to note that the monkeys underwent extensive preoperative training in order to master the strategy implementation task, so they were expert in executing the sequences of choices required to earn rewards efficiently and familiar with the reward schedules associated with the different choice stimuli. Thus, this task differs from studies of human decision-making that have determined the neural substrates of exploratory decision-making (Daw *et al.*, 2006) in that our monkeys are not faced with an ‘explore/exploit’ dilemma.

The impairment in strategy implementation performance after VLPFC lesions is striking in contrast to the lack of effect of these lesions in the reinforcer devaluation task. The ability to modify choice behavior in response to a change in the value of the food reward appears to be mediated exclusively by the orbital prefrontal cortex within the frontal lobe (Izquierdo *et al.*, 2004). Performance in this task is unaffected by lesions of VLPFC (present study), dorsolateral prefrontal cortex (Baxter *et al.*, 2008b) or anterior cingulate cortex (P.L. Croxson, A.S. Mitchell and M.G. Baxter, unpublished observation). This supports the view that different strategic and decision-making functions are localized within different regions of the prefrontal cortex (e.g. Daw *et al.*, 2006; Yoshida & Ishii, 2006). This is consistent with views of prefrontal cortex function that ascribe dissociable processing functions to different areas in other settings, for example stimulus selection in discrimination learning (Rogers *et al.*, 2000) and visual memory (Wilson *et al.*, 1993; Petrides *et al.*, 2002). Importantly, the present data show that different subregions within the prefrontal cortex are necessary for different types of choice behavior. The demonstration of differential activation of prefrontal subregions in the intact brain during different demands on decision-making and choice behavior does not imply that activity in these different regions is obligatory for these functions to occur normally.

One aspect of the experiment that bears comment is that the strategy implementation task was extensively trained preoperatively, whereas the reinforcer devaluation task was encountered only postoperatively. Thus, the order of training relative to surgery may be relevant to the dissociation between VLPFC and orbital prefrontal lesions. The sequence of training and testing was dictated mainly by practical factors. The training schedule used for the strategy implementation task took a considerable amount of time, and the time taken for normal monkeys to learn the task is highly variable; thus, it was not possible to examine lesion effects on acquisition of strategy implementation performance, which might have a different effect (Hampshire & Owen, 2006; Baxter *et al.*, 2008b). Also, because monkeys are exposed to highly-palatable (and less nutritive) rewards in the test apparatus in the reinforcer devaluation task, we were concerned about the effect training on this would have on performance of other tasks when we began these studies, because performance in other tasks is reinforced with standard banana pellets of more balanced nutritional content (rather than peanuts or chocolate). This latter concern turned out to be unfounded, so in future experiments we could combine preoperative assessments of reinforcer devaluation with performance of other tasks.

Our interpretation of the effect of VLPFC lesions on behavior in these tasks emphasizes a role in the representation and implementation of behavioral rules and strategies that use higher-order (for example, categorical) information about sensory stimuli to guide organized behavior, consistent with some views advanced from neuroimaging and neurophysiological studies (Bunge *et al.*, 2003). Of course, there are other views of how prefrontal cortex functions to guide behavior, such as working memory (Goldman-Rakic, 1996) or inhibitory control (Aron *et al.*, 2004). It is not clear that these theories of prefrontal function can fully account for the specific patterns of behavioral impairment that occur after VLPFC lesions. For example, the impairment in strategy implementation performance is better characterized as a disorganization of behavior and a failure to apply rules appropriately, rather than an impairment in inhibitory processing. This is because following VLPFC damage (or frontal-inferotemporal disconnection; Gaffan *et al.*, 2002) monkeys make sporadic responses when they should not and also fail to make them when they should. Also, a generalized impairment in inhibitory processing should have also impaired performance in the reinforcer devaluation task, which was not observed in monkeys with VLPFC lesions. The strategy task does not impose a substantial working memory load; although behavior must be sequenced in time for rewards to be earned efficiently, the relevant information is the response made (and the outcome) of the previous trial, which can be used to prospectively choose the category of stimulus to be selected on the next trial. In any case, VLPFC lesions do not impair working memory for visual objects (Rushworth *et al.*, 1997). Monkeys with frontal-inferotemporal disconnection can learn visual discrimination problems for delayed reinforcement (Browning & Gaffan, 2008), so the impairment caused by VLPFC lesions probably cannot be related to reduced value of delayed rewards postoperatively. Orbital prefrontal lesions also do not induce a general disruption of inhibitory behaviors related to reward processing (Baxter *et al.*, 2007; Chudasama *et al.*, 2007). The present observations may be more consistent with the view that the overall function of the prefrontal cortex is to evaluate and reject behaviorally maladaptive rules within different cognitive and behavioral domains (Wise *et al.*, 1996).

In other recent experiments on prefrontal cortex function, we have put forward the proposal that the specific function of the prefrontal cortex is in the representation of temporally complex serial events (Browning *et al.*, 2005, 2007; Browning & Gaffan, 2008; Wilson & Gaffan, 2008). The present data are consistent with this idea. The reinforcer devaluation task requires the monkey to be sensitive to the hedonic value of food in the mouth (rather than simply obtaining a reward or not), which is experienced only after the object has been chosen and the reward taken, whereas the strategy implementation task clearly requires the monkey to be sensitive to the sequence of choices among categories of visual objects. Thus, it is possible that both the VLPFC and orbital prefrontal cortex are involved in processing temporally complex serial events, but of different types, hedonic and visual. However, the present experiment was not designed specifically to test this idea.

There is a lack of localization of rule- and strategy-selective activity within the prefrontal cortex when this has been studied electrophysiologically (Wallis *et al.*, 2001; Genovesio *et al.*, 2005). In particular, Wallis *et al.* (2001) recorded from dorsolateral, ventrolateral and orbital prefrontal cortex and found similar distributions of neurons encoding abstract rules in these three areas. Similarly, reward-related information appears to be represented in multiple subregions of the prefrontal cortex (Watanabe, 1996; Leon & Shadlen, 1999; Tremblay & Schultz, 1999; cf. Wallis & Miller, 2003). Functional magnetic resonance imaging (fMRI) studies have reported differential activation for particular prefrontal regions, specifically dorsolateral and ventrolateral, in different aspects of rule-related behavior (Bunge *et al.*, 2003; Bunge, 2004). However, the failure to report activations in the orbital prefrontal cortex should perhaps be interpreted cautiously, because fMRI techniques have some limitations for detecting activation within orbital prefrontal regions (Stenger, 2006). In any case, these techniques cannot establish the necessity of particular prefrontal subregions for different aspects of decision-making or rule/strategy-guided behavior. The interconnectivity of VLPFC with other regions of the prefrontal cortex may enable information about rules and strategies that is stored and processed within the VLPFC to be reflected in the neural activity of these other regions of prefrontal cortex, which are not themselves required for performance of tasks that require the implementation of rules and strategies.

The observation that different prefrontal subregions mediate decision-making based on strategies and reward value indicates that distinct neural systems are necessary for different types of adaptive behavior. Said another way, particular aspects of adaptive behavior survive the ablation of specific subregions of the prefrontal cortex. This has several implications. First, the presence of double dissociations between effects of subregional lesions on different cognitive tasks may suggest a degree of parallel organization of some cognitive processes within the prefrontal cortex, rather than hierarchical. Some recent models of prefrontal cortex function have suggested that it is organized hierarchically in terms of anterior–posterior regions, perhaps based on level of abstraction (Badre & D’Esposito, 2007; Koechlin & Summerfield, 2007). Similarly, models of prefrontal function that postulate a network of prefrontal structures engaged by diverse cognitive demands (Duncan & Owen, 2000) would seem to predict that subregional lesions of the prefrontal cortex would produce mild deficits across multiple tasks, rather than double dissociations. Double dissociations of function between prefrontal cortical areas are consistent with models of prefrontal function that emphasize segregation of processing between different prefrontal areas, perhaps related to the degree of abstraction of behavioral rules (Wise *et al.*, 1996). Second, although task-relevant information may be represented throughout the prefrontal cortex, our data support the idea that only neurons in specific subregions of the prefrontal cortex are actually necessary for behaviors based on this information to be executed. This emphasizes the complementary nature of experimental techniques that interfere with brain function to techniques that record brain activity in neurologically intact organisms (Murray & Baxter, 2006). Finally, the preservation of specific aspects of adaptive behavior after disruption of particular subregions of the prefrontal cortex may suggest that cognitive rehabilitation after prefrontal damage may be achieved by focusing on capacities that rely more on relatively intact regions of the prefrontal cortex. Thus, these observations have substantial implications for understanding the functions of prefrontal cortex and for future investigations of the cognitive neurobiology of this region.

## Abbreviations

fMRI: functional magnetic resonance imaging
VLPFC: ventrolateral prefrontal cortex
